# Analysis of Toxic Components in Secondary Metabolites of Entomopathogenic Fungi *Clonostachys rosea* (Hipocreales: Bionectriaceae) from *Cephalcia chuxiongica* (Hymenoptera: Pamphiliidae)

**DOI:** 10.3390/microorganisms13102289

**Published:** 2025-10-01

**Authors:** Junjia Lu, Jian Liu, Huali Li, Yajiao Sun, Yunqiang Ma, Yonghe Li

**Affiliations:** 1 College of Landscape Architecture and Horticulture Sciences, Southwest Forestry University, Kunming 650224, China; lujunjia@swfu.edu.cn (J.L.); jian927520@163.com (J.L.);; 2 Key Laboratory of Forest Disaster Warning and Control of Yunnan Province, Southwest Forestry University, Kunming 650224, China; 3 College of Plant Protection, Yunnan Agricultural University, Kunming 650201, China

**Keywords:** *Cephalcia chuxiongica*, *Clonostachys rosea*, biological control, novel entomopathogens, bioactive components, toxicity assay

## Abstract

*Clonostachys rosea*, an entomopathogenic fungus that infects *Cephalcia chuxiongica*, is highly pathogenic and has significant potential for controlling the damage this pest causes to pine forests. To investigate the role of *C. rosea* secondary metabolites in fungal pathogenicity, we conducted toxicity assays using crude metabolite extracts. These assays evaluated the effects of different concentrations, larval developmental stages, and exposure methods on larval mortality. Gas chromatography–mass spectrometry (GC–MS) was subsequently employed to identify the chemical constituents of the crude extracts, and the toxicity of the identified compounds was assessed. The results showed that the crude extract at a concentration of 7.5 μg/mL exhibited the highest toxicity. Two hours post-treatment, the mortality rate of non-diapause larvae reached 65%, which was significantly higher than that of the diapause group. Moreover, contact toxicity was more lethal to *C. chuxiongica* larvae than oral exposure. A total of 23 compounds were identified from the crude extract, of which nine exhibited toxicity: 2-piperidone, hydrocinnamic acid, phenethyl alcohol, oleic acid, tryptophol, stearic acid methyl ester, myristic acid, dodecanoic acid, and benzeneacetic acid. Except for 2-piperidone, which showed low toxicity, the other eight compounds demonstrated notable contact toxicity against *C. chuxiongica* larvae. These findings confirm the insecticidal potential of *C. rosea* secondary metabolites and provide a valuable reference for the biological control of *C. chuxiongica* and other chewing insect pests.

## 1. Introduction

Because of its adaptability and wide distribution, pine has become a foundational species in China’s forest system. It not only constitutes an irreplaceable component of the national economy, but also plays a vital role in various sectors, including pharmaceutical research and development, forest chemical production, ecotourism, and construction engineering [1,2,3]. However, the larvae of *Cephalcia chuxiongica* (Hymenoptera: Pamphiliidae) pose a significant threat to pine forests, particularly to the needles of Pinaceae species. These larvae exhibit high host specificity, with a marked preference for species such as *Pinus yunnanensis*, *P. armandii*, and *Keteleeria evelyniana* (Gymnospermae: Pinaceae) [4,5]. The larvae initially spin silk to form webs at the base of pine needles, establishing protective shelters. They then sever needles, draw them into the webs for feeding, and continue to spin silk, constructing dense nests [6]. In severe infestations, the larvae can completely strip the trees of their needles, leaving only the petioles behind. This process results in large areas of scorched forest and significantly hinders pine growth [7]. The ecological damage caused by *C. chuxiongica* not only threatens forest health but also undermines the coordinated achievement of ecological, economic, and social benefits, which are central to China’s sustainable forestry development goals. Consequently, this species is a major target in China’s forestry pest management and poses a serious threat to forest sustainability [8,9].

Biological control has emerged as a leading strategy in modern pest management due to its ecological sustainability and effectiveness. By restoring natural ecological balance, it minimizes harm to non-target organisms and avoids the environmental and resistance-related issues commonly associated with chemical pesticides, thereby positioning itself as a preferred alternative. Currently, biological control efforts often focus on exploiting the insecticidal potential of entomopathogenic fungi (EPF), which exhibit strong virulence against a wide range of pest species. Under artificial culture conditions, many EPF strains are capable of producing diverse secondary metabolites with potent insecticidal properties [4,5,10]. These toxic metabolites play a critical role in the infection process and overall pathogenicity of EPF [11]. After penetrating the host cuticle, assisted by cuticle-degrading enzymes, EPF establish infection, proliferate by absorbing nutrients from the insect, and release toxic secondary metabolites that disrupt the host’s secretory and metabolic systems [5,12,13,14]. Numerous studies have shown that insect death during EPF infection is closely linked to these metabolic toxins. Pathological examinations of infected hosts often reveal that their death is primarily caused by the secondary toxic metabolites produced by the fungi [15,16,17]. Thus, toxic secondary metabolites are considered a major factor in the insecticidal activity of EPF.

*Clonostachys rosea* (Hipocreales: Bionectriaceae) is a widely used entomopathogenic fungus, capable of effectively controlling various pests by parasitizing the larvae, eggs, and female adults of nematodes as well as insects such as the mango hopper *Amritodus atkinsoni* (Hemiptera: Cicadelidae) and *Xylosandrus germanus* (Curculionidae: Scolytinae) [18,19,20,21]. This parasitism causes disease and mortality in the pests, thereby effectively preventing damage to plants. Consequently, *C. rosea* has become one of the primary research subjects in the study of biocontrol agents against plant pests. To date, at least 229 secondary metabolites have been isolated from *C. rosea*, mainly comprising 84 nitrogen-containing metabolites, 85 polyketides, 40 terpenoids, and 20 other compounds. These secondary metabolites exhibit significant cytotoxic and antibacterial activities, showing remarkable inhibitory effects on plant pathogens [22].

Given that high-virulence strains of *C. rosea* exhibit a rapid and potent lethal effect on *C. chuxiongica* larvae [23], this study aimed to investigate the insecticidal mechanism of its secondary metabolites. Fermentation broth of *C. rosea* was extracted and concentrated to obtain a crude metabolite extract. The effects of different concentrations, exposure durations, larval developmental stages, and administration methods on the mortality of *C. chuxiongica* larvae were systematically evaluated. In parallel, GC–MS was employed to identify the chemical constituents of the crude extracts. The insecticidal activities of the identified compounds were then assessed to determine their virulence against *C. chuxiongica* larvae. This integrated approach was designed to elucidate the mode of action of *C. rosea* secondary metabolites, identify the key pathogenic compounds involved, and provide a theoretical foundation for the development of biological control strategies against *C. chuxiongica*.

## 2. Materials and Methods

### 2.1. Experimental Materials

The entomopathogenic fungus *Clonostachys rosea* was isolated from the surface of naturally diseased and dead larvae of the sawfly *Cephalcia chuxiongica*. Detailed procedures for the isolation of this fungal strain, as well as preliminary research, have been previously published in our prior study [7,24]. Currently, *C. rosea* strain is preserved at the Laboratory of Forest Disaster Warning and Control, Yunnan Province.

The tested *Cephalcia chuxiongica* larvae used in this study were collected from the soil beneath pine forests in Beidaying Village, Qixing Township, Xundian County, Kunming City, Yunnan Province, China (25.30° N, 103.21° E; altitude: 1970 m). In this study, mature diapausing larvae and non-diapausing larvae at the 3rd–4th instars were collected in batches from March to November 2016, with approximately 500 larvae collected each batch. After collection, the larvae were quickly transferred to sterile soil under controlled laboratory conditions, with the relative humidity of the soil maintained at 30%, and fed pine needles regularly. Only healthy larvae that remained viable after 5 days of cultivation were selected for subsequent experiments. Larvae exhibiting damaged body surfaces, abnormal coloration (e.g., yellowing or darkening), fluid exudation, refusal to feed, sluggish movement, or curled immobility were excluded.

The culture medium used for fungal growth was Czapek-Dox Agar, composed of NaNO_3_ (2 g), K_2_HPO_4_ (1 g), KCl (0.5 g), MgSO_4_ (0.5 g), FeSO_4_ (0.01 g), and sucrose (30 g) per liter of distilled water. The medium was sterilized at 121 °C for 20 min.

### 2.2. Determination of Optimal Preparation Time for High-Virulence Fermentation Broth

A 0.1% (*v*/*v*) sterile Tween-80 solution was prepared by adding 5 mL of Tween 80 to 5000 mL of deionized water, followed by autoclaving at 115 °C for 30 min. After *C.s rosea* was cultured on solid medium plates for 20 days, aerial conidia produced on the medium surface were scraped into 50 mL sterile centrifuge tubes. An appropriate volume of sterile 0.1% (*v*/*v*) Tween-80 solution was added to each tube, and the mixture was vortexed for 3 min to form a conidial suspension. The suspension was filtered through six layers of sterile lens paper using a sterile funnel, and the filtrate was transferred to new sterile 50 mL centrifuge tubes. The concentration of the conidial suspension was standardized to 1 × 10^10^ conidia/mL and stored at 4 °C until use.

A conidial suspension of *C. rosea* at a concentration of 1.0 × 10^10^ conidia/mL was inoculated into the fermentation medium at a volume ratio of 5% (*v*/*v*) and incubated in a shaker at 25 °C and 200 rpm [24]. Fermentation broths were collected at 12, 24, 36, 48, 60, and 72 h post-inoculation. After centrifugation at 5000 rpm for 20 min, the supernatant was collected for bioassays. Non-diapause larvae were immersed for 1 min in fermentation broths maintained at 15 °C. Sterile water served as the control. Each treatment group consisted of 20 healthy larvae, with three biological replicates per group [25]. Treated larvae were transferred to 90-mm Petri dishes with controlled humidity and were regularly fed fresh *Pinus yunnanensis* needles. Larval mortality was recorded every 2 h.

### 2.3. Extraction of Metabolites from the Strain

*C. rosea* was cultured according to the method described in Section 2.2. Cultures were incubated until the optimal time point for producing high-virulence fermentation broth, as determined in Section 2.2. At this point, shaking incubation was terminated, and the fermentation broths were centrifuged at 5000 rpm for 20 min. The supernatant was reduced to half its original volume by rotary evaporation at 60 °C. Subsequently, twice the volume of 99% ethanol was added to the concentrated extract for alcohol precipitation, and the mixture was allowed to stand at room temperature for 24 h. After centrifugation at 4000 rpm for 20 min, the supernatant was collected, and ethanol was removed via rotary evaporation. Liquid–liquid extraction was performed with ethyl acetate (organic:aqueous = 2:1, *v*/*v*), repeated three times. The combined organic phase was concentrated and dried to yield the crude extract of fungal secondary metabolites.

### 2.4. Toxicity Determination of Crude Extracts from Strain Metabolites

#### 2.4.1. Toxicity Determination at Different Concentrations

The obtained crude extract was weighed, and the extraction yield was calculated using the following formula:

$$Yield\;(\%)=\frac{mass\;of\;crude\;extract}{original\;volume\;of\;fermentation\;broth}\times 100$$

Aqueous solutions of the crude metabolite extract were prepared at concentrations of 2.5 μg/mL, 5 μg/mL, and 7.5 μg/mL using sterile water. Non-diapause larvae were immersed in crude extract solutions at different concentrations for 1 min. Each treatment group contained 20 healthy larvae, with sterile water treatment serving as the control. Three biological replicates were performed for each treatment group. The post-treatment cultivation methods followed the procedures described in Section 2.2, and larval mortality was recorded every 2 h.

#### 2.4.2. Toxicity Determination on Larvae at Different Developmental Stages

The crude extract of fungal metabolites was diluted to the concentration exhibiting the highest virulence, as determined in Section 2.4.1. *C. chuxiongica* larvae in both diapause and non-diapause stages were immersed in the crude extract solution for 1 min, with sterile water used as the control treatment. For feeding, twenty *Pinus yunnanensis* needles (each approximately 3 cm in length) were placed in each Petri dish at the beginning of the experiment. Additional needles were provided based on the number of surviving larvae, at a rate of one needle per larva per supplementation.

#### 2.4.3. Determination of Virulence Under Different Toxicant Administration Methods

Group A (contact toxicity): Non-diapause *C. chuxiongica* larvae were starved for 3 h. The crude extract of fungal metabolites was diluted to the optimal virulence concentration determined in Section 2.4.1. At 15 °C, larvae were immersed in the aqueous crude extract solution for 1 min, with sterile water-treated larvae serving as controls. Each treatment consisted of 20 healthy larvae with three replicates. After treatment, larvae were transferred to humidity-controlled Petri dishes (30% relative humidity) and provided with 20 *P. yunnanensis* needles (3 cm in length) for feeding. Additional needles were supplemented based on the number of surviving larvae at a rate of one needle per larva per feeding. All larvae were maintained at room temperature and observed at 2-h intervals.

Group B (stomach toxicity): Non-diapause *C. chuxiongica* larvae were starved for 3 h. The crude extract was diluted to the optimal virulence concentration determined in Section 2.4.1. At 15 °C, *P. yunnanensis* needles were immersed in the extract for 30 min before being fed to the larvae, while needles soaked in sterile water for 30 min served as controls. Each treatment consisted of 20 healthy larvae with three biological replicates. After treatment, larvae were transferred to humidity-controlled Petri dishes and provided with additional needles based on survival rates (one needle per larva per feeding). All larvae were maintained at room temperature and observed at 2-h intervals.

### 2.5. Identification and Analysis of Chemical Components of Strain-Derived Metabolic Toxins

#### 2.5.1. Component Identification

The crude metabolite extract was filtered through a membrane filter and dissolved in chromatographic-grade methanol. Chemical composition analysis was performed using GC–MS with an Agilent 7890B-5977A system (Agilent Technologies, Santa Clara, CA, USA) equipped with an HP-5MS capillary column (30 m × 0.32 mm i.d. × 0.25 μm film thickness). The GC temperature program was as follows: initial temperature of 120 °C held for 2 min, ramped to 250 °C at 20 °C/min, and held for 20 min. Injection was performed in split mode with a split ratio of 10:1, and the inlet temperature was set at 250 °C. Mass spectrometry was performed using an electron impact (EI) ion source at 70 eV. The ion source temperature was set at 250 °C, the interface temperature at 280 °C, the quadrupole temperature at 150 °C, and the solvent delay was 3 min. Full-scan mode was used with a mass range of 20–650 *m*/*z*.

#### 2.5.2. Determination of the Insecticidal Activity of Toxic Compounds

The purified toxic compounds identified by GC-MS were dissolved as follows: except for phenethyl alcohol and 2-piperidone reference standards purchased from Tianjin Chemical Reagent Third Factory (Tianjin, China), which were directly dissolved in sterile water, all other reference compounds were first dissolved in dimethyl sulfoxide (DMSO) and then diluted with sterile water to concentrations of 0.05 mg/mL, 0.5 mg/mL, and 5 mg/mL. Non-diapause *C. chuxiongica* larvae were immersed in organic solutions at each concentration gradient for 1 min, with sterile water-treated larvae serving as controls. Each treatment consisted of 20 healthy larvae with three replicates. After treatment, larvae were transferred to humidity-controlled Petri dishes and provided with 20 *P. yunnanensis* needles (3 cm in length) for feeding. Observations were conducted at 2-h intervals, and needle supplementation was adjusted based on consumption rates. All larvae were maintained at room temperature, and mortality was recorded every 12 h.

The *C. rosea* conidial suspension (1.0 × 10^8^ conidia/mL) was compared with the optimal lethal concentration of toxic compounds to evaluate the lethal efficacy of the conidial suspension against *C. chuxiongica*. Each treatment included three biological replicates with 20 larvae per replicate (following the same rearing protocol as above).

### 2.6. Statistical Analysis

Statistical analysis was performed using GraphPad Prism 9.5 and SPSS 26.0. After verifying that all datasets met the assumptions of normality (Shapiro–Wilk test) and homoscedasticity (Levene’s test) with *p* > 0.05, a two-way analysis of variance (ANOVA) with a significance threshold of *p* < 0.05 was employed to evaluate inter-sample differences, followed by Tukey’s HSD test for pairwise comparisons. To further dissect significant interaction effects between specific factor levels, Šidák’s and Tukey’s multiple comparisons tests were systematically applied. Figures were prepared using GraphPad Prism 9.5.

## 3. Results and Analysis

### 3.1. Comparison of the Toxicity of Crude Extracts from Strain Metabolites

#### 3.1.1. Determination of Optimal Preparation Time for High-Virulence Fermentation Broth Analysis

Bioassay results evaluating the insecticidal activity of *Clonostachys rosea* fermentation broth against *Cephalcia chuxiongica* larvae indicated that the toxic potency of the broth peaked between 48 and 60 h post-inoculation. Based on this observation, the shaking incubation of the *C. rosea* fermentation culture was terminated after 60 h of liquid cultivation at 25 °C.

Following 60 h of submerged cultivation (25 °C, 200 rpm) of *C. rosea* in 6 L of medium, the mycelia were separated by filtration to obtain ~5.4 L of fermentation broth. The crude extract of *C. rosea* secondary metabolites, obtained through centrifugation, concentration, ethanol precipitation, liquid–liquid extraction, and drying, yielded 0.72 g of a dark brown, highly water-soluble paste, corresponding to an extraction efficiency of 133 mg/L.

#### 3.1.2. Toxicity Assay of Crude Extracts from the Strain’s Metabolites

##### Comparative Analysis of Toxic Potency at Different Concentrations

Analysis of covariance revealed that both the crude extract concentration and treatment time had extremely significant effects on the mortality of Cephalcia chuxiongica larvae (*p* < 0.0001). Furthermore, the interaction between the crude extract concentration and treatment time also exerted an extremely significant influence on larval mortality (*p* < 0.0001). Throughout the experimental observation period, the control group consistently showed 0% cumulative mortality.

**Figure 1:**
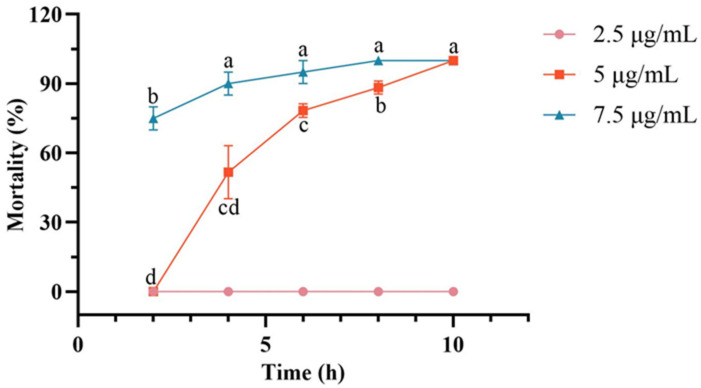
Changes in cumulative mortality of *Cephalcia chuxiongica* larvae over the treatment time gradient of 2–10 h under different concentrations: 2.5 μg/mL, 5 μg/mL, and 7.5 μg/mL. Different letters (a, b, c, d) above the data points indicate significant differences at the *p* < 0.05 level, where distinct letters represent statistically significant differences between groups.

Analysis of cumulative larval mortality across different treatment durations and concentrations revealed distinct patterns. At a concentration of 2.5 μg/mL, *C. chuxiongica* larvae exhibited 0% cumulative mortality at all assessed time points. At 5 μg/mL, treatment duration had a significant effect on mortality, showing a progressive increase over time. No mortality was observed at 2 h post-treatment, while cumulative mortality reached 51.7% at 4 h and 100% by 10 h. Statistically significant differences were observed between all pairwise comparisons of treatment durations with 2.5 μg/mL, except between the 6-h and 8-h groups and between the 8-h and 10-h groups. At 7.5 μg/mL, a similar time-dependent increase in cumulative mortality was observed. Mortality reached 65% at 2 h and 90% at 4 h post-treatment. Compared with 2.5 μg/mL, no notable differences in cumulative mortality of larvae were detected among the 4-h, 6-h, 8-h, and 10-h groups at 7.5 μg/mL; however, all these time points showed markedly higher mortality than the 2-h group. Complete mortality was achieved by 8 h post-treatment (Figure 1).

##### Comparative Analysis of Toxic Potency Against Larvae at Different Developmental Stages

Following a 1-min immersion in an aqueous solution of *C. rosea* crude extract at a concentration of 7.5 μg/mL, cumulative mortality of both non-diapause and diapause *C. chuxiongica* larvae was recorded. No mortality occurred in the control groups treated with sterile water, regardless of diapause status. In contrast, larvae exposed to the crude extract exhibited time-dependent increases in mortality.

**Figure 2:**
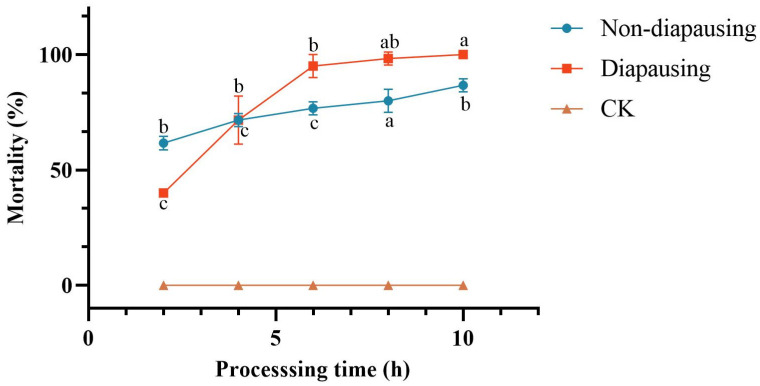
Changes in mortality of *Cephalcia chuxiongica* larvae over processing time across different developmental stages: Different letters (a, b, c, d) above the data points indicate significant differences at the *p* < 0.05 level, where distinct letters represent statistically significant differences between groups.

In non-diapause larvae, exposure to the crude extract resulted in a statistically significant increase in cumulative mortality over time. While no significant differences were observed between the 4-h group and other time points, mortality at 6 h post-treatment was significantly higher than that at 2 h (65%), reaching 90% by 10 h post-treatment (Figure 2).

In diapause larvae, exposure to the crude extract of *C. rosea* metabolites produced a cumulative mortality trend similar to that observed in the non-diapause group, characterized by a progressive increase with prolonged exposure duration. No significant differences in cumulative mortality were detected among the 6-h, 8-h, and 10-h treatment groups; however, mortality at these time points was significantly higher than at 4 h post-treatment. Cumulative mortality reached 95% at 6 h and 100% at 10 h. Additionally, mortality at 4 h post-treatment (76.67%) was significantly higher than at 2 h (41.67%).

For both non-diapause and diapause larvae, cumulative mortality increased progressively with treatment duration, although the rate and extent of change differed between the two groups). At 2 h post-treatment, the cumulative mortality of the non-diapause group (65%) was significantly higher than that of the diapause group (41.67%). By 4 h, mortality rates were comparable between the two groups. However, with extended exposure, the diapause group exhibited a more rapid increase in cumulative mortality, with significantly higher mortality than the non-diapause group at both 6 and 8 h post-treatment. By 10 h, although the cumulative mortality of the diapause group exceeded that of the non-diapause group, the difference was not statistically significant.

##### Determination of Toxic Potency Under Different Toxicant Administration Methods

Non-diapause *C. chuxiongica* larvae were selected as test subjects and exposed to two treatment modalities: contact toxicity and oral toxicity. Throughout the experimental period, both treatments resulted in a progressive increase in cumulative larval mortality, while no mortality was observed in the control group.

**Figure 3:**
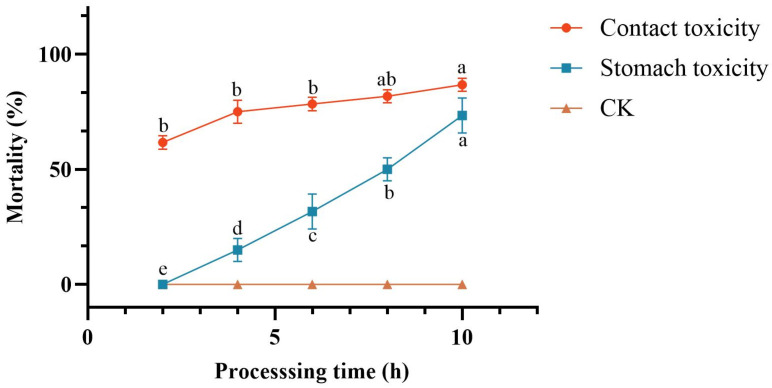
Comparison of lethality rates between contact and oral toxicity treatments. Different letters (a, b, c, d) above the data points indicate significant differences at the *p* < 0.05 level, where distinct letters represent statistically significant differences between groups.

In *C. chuxiongica* larvae treated with the crude extract via the contact toxicity method, cumulative mortality increased significantly over time. Although no significant differences were observed between the 4-h group and other time points, cumulative mortality at 6 h post-treatment was significantly higher than at 2 h (65%), reaching 90% by 10 h post-treatment (Figure 3).

In *C. chuxiongica* larvae treated with the crude extract via the oral toxicity method, cumulative mortality also exhibited a significant upward trend with increasing exposure duration. No mortality was observed at 2 h post-treatment; however, larval mortality increased progressively over time, with significant differences in cumulative mortality recorded at each subsequent 2-h interval.

When comparing larval mortality under the two treatment methods (contact and oral toxicity), cumulative mortality exhibited a time-dependent increase in both groups; however, the rate and magnitude of mortality differed. Across all time points prior to 10 h, cumulative mortality under contact toxicity treatment was consistently and significantly higher than that under oral toxicity treatment. At 10 h post-treatment, although the cumulative mortality in the contact toxicity group (90%) remained higher than that in the oral toxicity group (73.3%), the difference was not statistically significant.

### 3.2. Identification of Chemical Constituents in Crude Extracts from Strain Metabolites

The chemical constituents of the crude extract derived from *C. rosea* secondary metabolites were analyzed using GC–MS. Chromatographic peaks were primarily detected within the retention time range of 5.348–23.980 min, yielding a total of 65 peaks. Based on GC–MS analysis and mass spectral library matching, 65 components were identified, of which 23 exhibited a match score ≥ 30% (Table 1). These 23 components were further screened using the Chemical Substances Toxicity Database (accessed on 10 December 2024, http://www.drugfuture.com/toxic/search.aspx), resulting in the identification of 9 major toxic compounds: phenethyl alcohol (7.27% of total toxic content), 2-piperidone (5.13%), benzeneacetic acid (3.13%), hydrocinnamic acid (5.6%), dodecanoic acid (1.43%), myristic acid (5.5%), tryptophol (4.2%), stearic acid methyl ester (1.1%), and oleic acid (66.63%).

### 3.3. Insecticidal Activity Assay of Toxic Compounds

#### 3.3.1. Determination of the Insecticidal Activity of Oleic Acid

Oleic acid at a concentration of 0.05 mg/mL exhibited no lethal effect on *C. chuxiongica* larvae. However, larval mortality increased with higher concentrations of oleic acid. At 0.5 mg/mL, more than 50% of larvae died within 108 h post-treatment, with cumulative mortality reaching 81.67% by 180 h and a calculated median lethal time (LT_50_) of 77 h. At 5 mg/mL, nearly 50% mortality was observed within the first 12 h, and complete mortality (100%) was achieved within 96 h of treatment (Table 2).

#### 3.3.2. Determination of the Insecticidal Activity of Tryptophol

As shown in Table 3, tryptophol at a concentration of 0.05 mg/mL exhibited no toxic effect on *C. chuxiongica* larvae. However, mortality increased with higher tryptophol concentrations. At 0.5 mg/mL, cumulative mortality reached 75.00% after 180 h of treatment, with an LT_50_ of 83.3 h. At 5 mg/mL, 50% of the larvae died within 12 h, and mortality rose to 98.33% by 180 h.

#### 3.3.3. Determination of the Insecticidal Activity of Stearic Acid Methyl Ester

As shown in Table 4, stearic acid methyl ester at a concentration of 0.05 mg/mL exhibited negligible toxicity against *C. chuxiongica* larvae. In contrast, higher concentrations showed clear toxic effects, with mortality increasing in a concentration-dependent manner. At 0.5 mg/mL, cumulative mortality reached 75.00% after 180 h of treatment, with an LT_50_ of 110 h. At 5 mg/mL, more than 50% mortality was observed within 48 h, and cumulative mortality reached 98.33% by 180 h.

#### 3.3.4. Determination of the Insecticidal Activity of Myristic Acid

As shown in Table 5, myristic acid at a concentration of 0.05 mg/mL exhibited negligible toxicity against *C. chuxiongica* larvae. In contrast, higher concentrations demonstrated significant toxicity, with mortality increasing in a concentration-dependent manner. At 0.5 mg/mL, cumulative mortality reached 73.33% after 180 h of treatment, with an LT_50_ of 80 h. At 5 mg/mL, over 50% mortality was observed within 60 h, and complete mortality (100%) was achieved by 144 h.

#### 3.3.5. Determination of the Insecticidal Activity of Dodecanoic Acid

As shown in Table 6, dodecanoic acid at a concentration of 0.05 mg/mL exhibited negligible toxicity against *C. chuxiongica* larvae. In contrast, higher concentrations displayed clear toxic effects, with mortality increasing in a concentration-dependent manner. At 0.5 mg/mL, cumulative mortality reached 60.00% after 180 h of treatment, with an LT_50_ of 99.3 h. At 5 mg/mL, over 50% of the larvae died within 48 h, and complete mortality (100%) was achieved by 144 h.

#### 3.3.6. Determination of the Insecticidal Activity of 2-Piperidone

As shown in Table 7, 2-piperidone exhibited no detectable toxicity against *C. chuxiongica* larvae at any of the tested concentrations.

#### 3.3.7. Determination of the Insecticidal Activity of Phenethyl Alcohol

Phenethyl alcohol at a concentration of 0.05 mg/mL exhibited no toxic effect on *C. chuxiongica* larvae. However, higher concentrations displayed clear toxicity, with mortality increasing in a concentration-dependent manner. At 0.5 mg/mL, cumulative mortality reached 76.67% after 180 h of treatment, with an LT_50_ of 114 h. At 5 mg/mL, more than 50% of larvae died within 84 h of exposure (Table 8).

#### 3.3.8. Determination of the Insecticidal Activity of Benzeneacetic Acid

Benzeneacetic acid at a concentration of 0.05 mg/mL exhibited no toxicity against *C. chuxiongica* larvae. In contrast, higher concentrations showed clear toxic effects, with mortality increasing in a concentration-dependent manner. At 0.5 mg/mL, cumulative mortality reached 70.00% after 180 h of treatment, with an LT_50_ of 130.7 h. At 5 mg/mL, more than 50% mortality was observed within 60 h, and complete mortality (100%) was achieved by 84 h (Table 9).

#### 3.3.9. Determination of the Insecticidal Activity of Hydrocinnamic Acid

Hydrocinnamic acid at a concentration of 0.05 mg/mL exhibited no toxicity against *C. chuxiongica* larvae. However, higher concentrations demonstrated clear toxic effects, with mortality increasing in a concentration-dependent manner. At 0.5 mg/mL, cumulative mortality reached 80.00% after 180 h of treatment, with an LT_50_ of 92 h (Table 10).

#### 3.3.10. Comparison of the Toxicity Between Identified Compounds and Conidial Suspensions

**Figure 4:**
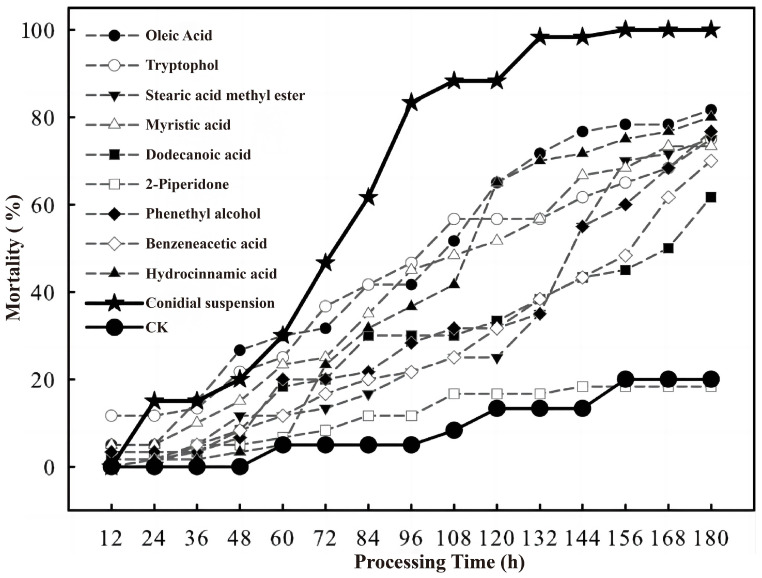
Comparison of mortality of *Cephalcia chuxiongica* larvae exposed to different toxic compounds (oleic acid, tryptophol, stearic acid methyl ester, myristic acid, dodecanoic acid, 2-piperidone, phenethyl alcohol, benzeneacetic acid and hydrocinnamic acid), conidial suspension of *Clonostachys rosea*, and sterile water treatment (CK) at different treatment times (12–180 h).

A comparative analysis of insecticidal activity was conducted between each identified toxic compound at a concentration of 0.5 mg/mL and a *C. rosea* conidial suspension at 1.0 × 10^8^ conidia/mL, as shown in Figure 4. The results indicated that the 2-piperidone treatment group exhibited no significant difference from the control group, confirming its lack of toxicity. In contrast, the remaining eight compounds—hydrocinnamic acid, phenethyl alcohol, oleic acid, tryptophol, stearic acid methyl ester, myristic acid, dodecanoic acid, and benzeneacetic acid—demonstrated insecticidal activity against *C. chuxiongica* larvae. However, their toxic effects were markedly lower than those obs erved with the *C. rosea* conidial suspension. Notably, the difference in lethality between the conidial suspension and each of the toxic compounds became highly significant after 60 h of treatment. For the analysis of the lethal ability of *C. rosea* against *C. chuxiongica*, please refer to Supplementary Materials Table S1.

## 4. Discussion

Analysis of *Cephalcia chuxiongica* larvae mortality exposed to *Clonostachys rosea* crude extracts showed that a 7.5 μg/mL aqueous solution was most toxic: 65% died within 2 h, and 100% after 8 h. A previous study on *Beauveria brongniartii* (Ascomycota: Cordycipitaceae) against *Dendrolimus tabulaeformis* (Lepidoptera: Lasiocampidae) reported that the cumulative mortality rate of *D. tabulaeformis* was 71.3% after 7 days of treatment with the highest toxicity concentration (550 μg/mL) in the toxicity assay of crude extracts of metabolic toxins from *B. brongniartii* against *D. tabulaeformis* [26]. In contrast, *C. rosea*’s crude secondary metabolite extract was more toxic, showing advantages in low effective concentration, rapid action, and high efficacy, supporting its potential as a better alternative to *B. brongniartii*-based bioinsecticides.

Furthermore, the larvae exhibited pronounced twisting behavior when exposed to the crude extract, which is consistent with the discoveries of Baggio-Deible [27] and Haiyang Wang [28]. These findings are consistent with previous studies. A comparison of the toxicity of the crude extract of the strain’s metabolites against larvae at various developmental stages revealed that it is toxic to both diapausing and non-diapausing *C. chuxiongica* larvae. The toxicity of the substance was more potent against non-diapause larvae within four hours of treatment than it was against diapause larvae. However, the toxicity of the substance against diapause larvae became more potent after four hours of treatment. The physiological status of the larvae (metabolic activity, barrier function, detoxification ability) and the mechanism of action of the toxin are the primary factors contributing to the time-dependent difference in toxicity. The toxin’s absorption and action are expedited by the high metabolic activity of non-diapause larvae in the early stage, whereas the low metabolic state of diapause larvae results in long-term accumulation and slow toxin clearance, ultimately leading to stronger toxicity in the later stage [28,29,30,31]. This phenomenon also implies that application timing and dosage of fungal crude extracts should be tailored to different insect developmental stages, to enhance biological control efficiency.

In addition, the results showed that contact toxicity caused more larval deaths than oral toxicity in *C. chuxiongica*. The main reason for the higher efficiency of the contact toxicity method may be that it bypasses the defensive barrier of the larval digestive tract, utilizing the permeability of the cuticle to achieve rapid invasion and action of toxins. In contrast, the oral toxicity method is limited by feeding behavior, digestive barriers, and detoxification mechanisms, reducing toxin efficiency [32,33]. This suggests that in biological control of this pest, prioritizing contact-type fungal preparations, such as conidial suspensions and metabolites capable of penetrating the body wall, may more effectively enhance control efficacy.

During the experiment, it was observed that *C. chuxiongica* larvae in Petri dishes died and exhibited body stiffening even without feeding on pine needles treated with the crude extract of *C. rosea* metabolites. This phenomenon may be attributed to the volatility of certain *C. rosea* metabolites, which can exert toxic effects through fumigation. In the present study, GC–MS analysis of the crude extract of *C. rosea* secondary metabolites identified nine toxic compounds. Although these compounds exhibited lethal effects on *C. chuxiongica* larvae at a concentration of 0.5 mg/mL, their toxicity was lower than that of the *C. rosea* conidial suspension (1.0 × 10^8^ conidia/mL). Shichuang Ma [34] evaluated the insecticidal activity of toxic compounds against *Myzus persicae*, *Aphis pomi*, *Brevicoryne brassicae* (Hemiptera: Aphididae), and *Bemisia tabaci* (Hemiptera: Aleyrodidae), and found that the derivatives exhibited notable contact and systemic effects. Similarly, Cecilia Labbé [35] isolated 2-phenylethyl acetate and 2-phenylethyl benzoate from the extract of *Balantiopsis cancellata* (Bryophyta: Balantiopsaceae), a Chilean plant species, and identified their antifeedant activity against *Spodoptera frugiperda* (Lepidoptera: Noctuidae). These previous findings collectively support the conclusion that toxic compounds induce rapid insect mortality through disruption of insect-specific metabolic pathways and physiological mechanisms. Building on this foundation, the toxin action mechanism elucidated in the present study provides a critical theoretical basis for developing novel green pesticides with high target specificity and superior environmental compatibility, thereby offering substantial practical value for advancing integrated pest management (IPM) and promoting sustainable agricultural practices.

Previous studies have demonstrated that toxic compounds can rapidly induce insect mortality by disrupting insect-specific metabolic pathways and other physiological mechanisms. However, Moraes [36] compared the efficacy of *Bacillus thuringiensis* (Bacillota: Bacillaceae) formulations with that of chemical insecticides, such as diphenylurea and deltamethrin, against *Plutella xylostella* (Lepidoptera: Plutellidae). The findings indicated that *B. thuringiensis* exhibited superior control over third-instar larvae compared to the chemical agents. Similarly, Jiang Shuai et al. [37] conducted toxicity assays using crude toxin extracts from *Amanita muscaria* (Basidiomycota: Amanitaceae) and *Amanita verna* (Basidiomycota: Amanitaceae) and confirmed their insecticidal activity against *Gryllotalpa unispina* (Orthoptera: Gryllotalpidae). These studies highlight the promising potential of microbial and natural compound-based agents in pest management strategies. Parajuli [38] also reported that fungal biopesticides, including *Beauveria bassiana* (Ascomycota: Cordycipitaceae) *and Metarhizium anisopliae* (Ascomycota: Clavicipitaceae), are highly effective in controlling a wide range of insect pests, such as aphids, whiteflies, and locusts. These fungal agents function through mechanisms such as cuticle penetration and the production of toxic secondary metabolites. Compared to conventional chemical pesticides, fungal biopesticides are more environmentally friendly and represent a sustainable alternative for pest management. Thus, microbial formulations offer broader modes of action and present a promising strategy for advancing sustainable agricultural practices.

Investigating the crude extract of insect-derived secondary metabolites from *C. rosea* fermentation broth facilitates the elucidation of its insecticidal pathways and mechanisms of action. Such studies provide a theoretical basis for the application of *C. rosea* in biological pest control and contribute to assessing its safety for humans and animals. Among the identified metabolites, benzeneacetic acid—an important organic intermediate—holds broad application potential, particularly in the pharmaceutical and agrochemical industries [39,40,41]. However, due to the complexity of its synthetic routes, it remains a relatively scarce resource in the domestic pharmaceutical market. Additionally, stearic acid methyl ester is widely used in the synthesis of surfactants, lubricants, and other industrial chemicals [42], while tryptophol serves as a valuable reagent in organic synthesis [43]. Given that *C. rosea* can stably produce these metabolites through a fermentation process that is both scalable and easily regulated, it presents a sustainable and renewable source of raw materials for industrial applications, offering new opportunities for green biomanufacturing in related sectors.

Based on the above findings, a canopy spraying system is recommended for forest application. This system utilizes the fumigation effect of volatile compounds to penetrate pine needle layers, enabling three-dimensional control of concealed pests. Subsequent research will focus on optimizing large-scale fermentation processes to increase target compound yields, and verifying sustained control efficacy at different concentration gradients through field trials. This green pest control system not only provides a new solution for pine forest IPM, but also pioneers a sustainable development model integrating biological control with green manufacturing.

## 5. Conclusions

By extracting and purifying the crude metabolites of *Clonostachys rosea*, toxicity assays were conducted on *Cephalcia chuxiongica* larvae across different concentrations, developmental stages, and exposure methods. The results indicated that the crude extract at a concentration of 7.5 μg/mL exhibited the highest insecticidal activity, achieving 100% larval mortality within 8 h post-treatment. When larvae at different developmental stages were exposed to the same concentration, the non-diapause group showed a significantly higher mortality rate (65%) at 2 h compared to the diapause group (41.67%). However, after 10 h of exposure, cumulative mortality reached 90% in the non-diapause group, while 100% mortality was observed in the diapause group, indicating a temporal difference in susceptibility. In terms of exposure methods, contact toxicity was found to be more effective than oral toxicity in inducing larval death. The metabolic components of the *C. rosea* crude extract were identified using GC-MS, resulting in the detection of 23 compounds, among which 9 exhibited significant toxicity. Except for 2-piperidone, which showed low toxicity, eight compounds—hydrocinnamic acid, phenethyl alcohol, oleic acid, tryptophol, stearic acid methyl ester, myristic acid, dodecanoic acid, and benzeneacetic acid—demonstrated contact toxicity against *C. chuxiongica* larvae. Among these, myristic acid exhibited the strongest insecticidal activity, although its efficacy was still inferior to that of the *C. rosea* conidial suspension at 1.0 × 10^8^ conidia/mL.

This study confirms that the secondary metabolites of *C. rosea* exert lethal effects on *C. chuxiongica*, highlighting their considerable potential for pest management. These findings provide a robust theoretical foundation for the development of novel biocontrol strategies and the enhancement of pest control precision. In subsequent investigations, we aim to identify the specific molecular targets of *C. rosea* secondary metabolites in *C. chuxiongica*, with the goal of elucidating the underlying molecular and physiological mechanisms governing their insecticidal activity. Additionally, we will optimize the formulation and application parameters of this biocontrol agent to minimize unintended ecological impacts on non-target organisms, ensuring its sustainable integration into forest pest management strategies.

## Figures and Tables

**Table 1:** Compounds detected in the crude extract of *Clonostachys rosea* using Gas Chromatography-Mass Spectrometry (GC-MS) analysis.

Number	Peak Time	Molecular Formula	IUPAC Name	Content (%)	Match Score (%)
1	5.348	C_8_H_10_O	Phenethyl alcohol	2.18	93
2	6.146	C_5_H_9_NO	2-Piperidone	1.54	96
3	6.894	C_8_H_8_O_2_	Benzeneacetic acid	0.94	93
4	7.319	C_10_H_12_O_2_	Methyl 3-phenylpropionate	0.17	93
5	7.844	C_9_H_10_O_2_	Hydrocinnamic acid	1.68	96
6	8.707	C_8_H_10_O_2_	2-(4-Hydroxyphenyl)ethanol	0.95	87
7	9.714	C_12_H_24_O_2_	Dodecanoic acid	0.43	99
8	11.002	C_14_H_28_O_2_	Myristic acid	1.65	99
9	11.139	C_10_H_11_NO	Tryptophol	1.26	90
10	11.362	C_15_H_30_O_2_	Pentadecanoic acid	0.61	99
11	11.829	C_17_H_32_O_2_	(Z)-Methyl hexadec-9-enoate	0.20	99
12	11.923	C_17_H_34_O_2_	Methyl palmitate	1.18	99
13	12.009	C_16_H_30_O_2_	cis-9-Hexadecenoic acid	3.18	99
14	12.103	C_16_H_32_O_2_	Palmitic acid	14.9	99
15	12.182	C_16_H_22_O_4_	Dibutyl phthalate	1.97	97
16	12.635	C_17_H_34_O_2_	Heptadecanoic acid	0.11	87
17	12.793	C_21_H_42_	10-heneicosene	0.27	98
18	13.038	C_19_H_38_O_2_	Stearic acid methyl ester	0.33	98
19	13.139	C_18_H_34_O_2_	Oleic acid	19.99	99
20	13.268	C_18_H_36_O_2_	Stearic acid	1.13	99
21	13.685	C_18_H_32_O_2_	Linoleic acid	1.31	97
22	14.678	C_18_H_34_O_2_	(6Z)-6-Octadecenoic acid	0.22	87
23	21.707	C_22_H_43_NO	(Z)-Docos-13-enamide	0.44	94

**Table 2:** Mortality of Non-diapause *Cephalcia chuxiongica* Larvae Exposed to Different Concentrations of Oleic Acid.

Treatment Time (h)	CK	0.05 mg/mL	0.5 mg/mL	5 mg/mL
	Mean ± SD			
12	0.00 ± 0.00	0.00 ± 0.00	5.00 ± 4.08	43.33 ± 15.9
24	0.00 ± 0.00	0.00 ± 0.00	5.00 ± 4.08	65.00 ± 5.77
36	0.00 ± 0.00	0.00 ± 0.00	15.00 ± 10.8	65.00 ± 5.77
48	0.00 ± 0.00	0.00 ± 0.00	26.67 ± 6.24	78.33 ± 1.67
60	5.00 ± 2.89	0.00 ± 0.00	30.00 ± 7.07	90.0 ± 2.89
72	5.00 ± 2.89	1.67 ± 1.67	31.67 ± 8.5	95.00 ± 2.89
84	5.00 ± 2.89	3.33 ± 1.67	41.67 ± 4.71	98.33 ± 1.67
96	5.00 ± 2.89	3.33 ± 1.67	41.67 ± 4.71	100.00 ± 0.00
108	8.33 ± 6.01	6.67 ± 1.67	51.67 ± 4.71	100.00 ± 0.00
120	10.00 ± 2.89	6.67 ± 1.67	65.00 ± 4.08	100.00 ± 0.00
132	10.00 ± 2.89	10.00 ± 2.89	71.67 ± 4.71	100.00 ± 0.00
144	10.00 ± 2.89	10.00 ± 2.89	76.67 ± 7.07	100.00 ± 0.00
156	10.00 ± 2.89	10.00 ± 2.89	78.33 ± 8.5	100.00 ± 0.00
168	20.00 ± 4.08	10.00 ± 2.89	78.33 ± 8.5	100.00 ± 0.00
180	20.00 ± 4.08	20.00 ± 4.08	81.67 ± 9.43	100.00 ± 0.00

**Table 3:** Mortality of Non-diapause *Cephalcia chuxiongica* Larvae Exposed to Different Concentrations of Tryptophol.

Treatment Time (h)	CK	0.05 mg/mL	0.5 mg/mL	5 mg/mL
	Mean ± SD			
12	0.00 ± 0.00	0.00 ± 0.00	11.67 ± 6.24	50.00 ± 5.00
24	0.00 ± 0.00	0.00 ± 0.00	11.67 ± 6.24	53.33 ± 4.41
36	0.00 ± 0.00	0.00 ± 0.00	13.33 ± 8.50	53.33 ± 4.41
48	0.00 ± 0.00	1.67 ± 1.67	21.67 ± 2.36	60.00 ± 0.00
60	5.00 ± 2.89	3.33 ± 1.67	25.00 ± 4.08	63.33 ± 1.67
72	5.00 ± 2.89	5.00 ± 0.00	36.67 ± 10.27	68.33 ± 4.41
84	5.00 ± 2.89	5.00 ± 2.89	41.67 ± 9.43	68.33 ± 4.41
96	5.00 ± 2.89	6.67 ± 1.67	46.67 ± 9.28	85.00 ± 2.89
108	8.33 ± 6.01	10.00 ± 2.89	51.67 ± 4.71	86.67 ± 1.67
120	10.00 ± 2.89	13.33 ± 4.41	56.67 ± 4.71	86.67 ± 1.67
132	10.00 ± 2.89	15.00 ± 2.89	66.67 ± 7.64	88.33 ± 1.67
144	10.00 ± 2.89	15.00 ± 2.89	68.33 ± 5.77	88.33 ± 1.67
156	10.00 ± 2.89	15.00 ± 2.89	65.00 ± 5.77	90.00 ± 2.89
168	20.00 ± 4.08	15.00 ± 2.89	73.33 ± 2.89	95.00 ± 2.89
180	20.00 ± 4.08	15.00 ± 2.89	75.00 ± 2.89	98.33 ± 1.67

**Table 4:** Mortality of Non-diapause *Cephalcia chuxiongica* Larvae Exposed to Different Concentrations of Stearic Acid.

Treatment Time (h)	CK	0.05 mg/mL	0.5 mg/mL	5 mg/mL
	Mean ± SD			
12	0.00 ± 0.00	0.00 ± 0.00	0.00 ± 0.00	10.00 ± 5.77
24	0.00 ± 0.00	0.00 ± 0.00	0.00 ± 0.00	40.00 ± 8.66
36	0.00 ± 0.00	0.00 ± 0.00	5.00 ± 4.08	51.67 ± 8.33
48	0.00 ± 0.00	0.00 ± 0.00	11.67 ± 2.36	53.33 ± 9.28
60	5.00 ± 2.89	0.00 ± 0.00	11.67 ± 2.36	58.33 ± 9.28
72	5.00 ± 2.89	0.00 ± 0.00	13.33 ± 4.71	65.00 ± 5.77
84	5.00 ± 2.89	3.33 ± 1.67	16.67 ± 4.71	73.33 ± 1.67
96	5.00 ± 2.89	5.00 ± 0.00	21.67 ± 4.71	80.00 ± 5.77
108	8.33 ± 6.01	6.67 ± 1.67	25.00 ± 7.07	80.00 ± 5.77
120	10.00 ± 2.89	6.67 ± 1.67	25.00 ± 7.07	86.67 ± 1.67
132	10.00 ± 2.89	8.33 ± 3.33	35.00 ± 7.07	88.33 ± 1.67
144	10.00 ± 2.89	10.00 ± 2.89	55.00 ± 8.16	93.33 ± 4.41
156	10.00 ± 2.89	10.00 ± 2.89	70.00 ± 7.07	95.00 ± 2.89
168	20.00 ± 4.08	13.33 ± 4.41	71.67 ± 6.24	98.33 ± 1.67
180	20.00 ± 4.08	15.00 ± 2.89	75.00 ± 4.08	98.33 ± 1.67

**Table 5:** Mortality of Non-diapause *Cephalcia chuxiongica* Larvae Exposed to Different Concentrations of Myristic Acid.

Treatment Time (h)	CK	0.05 mg/mL	0.5 mg/mL	5 mg/mL
	Mean ± SD			
12	0.00 ± 0.00	0.00 ± 0.00	5.00 ± 0.00	10.00 ± 2.89
24	0.00 ± 0.00	0.00 ± 0.00	5.00 ± 0.00	25.00 ± 5.00
36	0.00 ± 0.00	0.00 ± 0.00	10.00 ± 4.08	35.00 ± 7.64
48	0.00 ± 0.00	0.00 ± 0.00	15.00 ± 4.08	35.00 ± 7.64
60	5.00 ± 2.89	0.00 ± 0.00	23.33 ± 4.71	53.33 ± 1.67
72	5.00 ± 2.89	0.00 ± 0.00	25.00 ± 4.08	60.00 ± 2.89
84	5.00 ± 2.89	1.67 ± 1.67	35.00 ± 4.08	71.67 ± 9.28
96	5.00 ± 2.89	3.33 ± 1.67	45.00 ± 4.08	86.67 ± 3.33
108	8.33 ± 6.01	6.67 ± 1.67	48.33 ± 2.36	90.00 ± 5.00
120	10.00 ± 2.89	8.33 ± 3.33	51.67 ± 2.36	95.00 ± 2.89
132	10.00 ± 2.89	10.00 ± 5.00	56.67 ± 4.71	96.67 ± 3.33
144	10.00 ± 2.89	11.67 ± 4.41	66.67 ± 7.64	100.00 ± 0.00
156	10.00 ± 2.89	11.67 ± 4.41	68.33 ± 1.67	100.00 ± 0.00
168	20.00 ± 4.08	13.33 ± 10.93	73.33 ± 6.24	100.00 ± 0.00
180	20.00 ± 4.08	13.33 ± 10.93	73.33 ± 6.24	100.00 ± 0.00

**Table 6:** Mortality of Non-diapause Cephalcia chuxiongica Larvae Exposed to Different Concentrations of Dodecanoic Acid.

Treatment Time (h)	CK	0.05 mg/mL	0.5 mg/mL	5 mg/mL
	Mean ± SD			
12	0.00 ± 0.00	0.00 ± 0.00	1.67 ± 2.36	25.00 ± 8.66
24	0.00 ± 0.00	0.00 ± 0.00	1.67 ± 2.36	33.33 ± 4.41
36	0.00 ± 0.00	0.00 ± 0.00	3.33 ± 2.36	31.67 ± 8.82
48	0.00 ± 0.00	0.00 ± 0.00	8.33 ± 8.50	53.33 ± 18.78
60	5.00 ± 2.89	0.00 ± 0.00	18.33 ± 2.36	60.00 ± 16.07
72	5.00 ± 2.89	0.00 ± 0.00	18.33 ± 2.36	61.67 ± 17.40
84	5.00 ± 2.89	1.67 ± 1.67	20.00 ± 4.08	68.33 ± 11.67
96	5.00 ± 2.89	3.33 ± 1.67	31.67 ± 1.67	81.67 ± 6.01
108	8.33 ± 6.01	3.33 ± 1.67	31.67 ± 1.67	91.67 ± 1.67
120	10.00 ± 2.89	5.00 ± 2.89	35.00 ± 3.35	96.67 ± 1.67
132	10.00 ± 2.89	10.00 ± 2.89	38.33 ± 13.12	96.67 ± 1.67
144	10.00 ± 2.89	10.00 ± 2.89	43.33 ± 6.24	96.67 ± 1.67
156	10.00 ± 2.89	11.67 ± 2.36	48.33 ± 4.08	100.00 ± 0.00
168	20.00 ± 4.08	11.67 ± 2.36	55.00 ± 1.67	100.00 ± 0.00
180	20.00 ± 4.08	20.00 ± 4.08	60.00 ± 1.67	100.00 ± 0.00

**Table 7:** Mortality of Non-diapause Cephalcia chuxiongica Larvae Exposed to Different Concentrations of 2-Piperidinone.

Treatment Time (h)	CK	0.05 mg/mL	0.5 mg/mL	5 mg/mL
	Mean ± SD			
12	0.00 ± 0.00	0.00 ± 0.00	0.00 ± 0.00	0.00 ± 0.00
24	0.00 ± 0.00	0.00 ± 0.00	1.67 ± 2.36	0.00 ± 0.00
36	0.00 ± 0.00	0.00 ± 0.00	5.00 ± 0.00	0.00 ± 0.00
48	0.00 ± 0.00	0.00 ± 0.00	5.00 ± 0.00	0.00 ± 0.00
60	5.00 ± 2.89	0.00 ± 0.00	6.67 ± 2.36	1.67 ± 1.67
72	5.00 ± 2.89	0.00 ± 0.00	8.33 ± 2.36	1.67 ± 1.67
84	5.00 ± 2.89	1.67 ± 1.67	11.67 ± 2.36	3.33 ± 1.67
96	5.00 ± 2.89	3.33 ± 1.67	11.67 ± 2.36	10.00 ± 2.89
108	8.33 ± 6.01	3.33 ± 1.67	16.67 ± 2.36	10.00 ± 2.89
120	10.00 ± 2.89	3.33 ± 1.67	16.67 ± 4.71	10.00 ± 2.89
132	10.00 ± 2.89	3.33 ± 1.67	16.67 ± 4.71	10.00 ± 2.89
144	10.00 ± 2.89	3.33 ± 1.67	20.00 ± 0.01	10.00 ± 2.89
156	10.00 ± 2.89	5.00 ± 2.89	20.00 ± 0.01	11.67 ± 2.36
168	20.00 ± 4.08	5.00 ± 2.89	21.67 ± 1.67	11.67 ± 2.36
180	20.00 ± 4.08	8.33 ± 6.01	21.67 ± 1.67	16.67 ± 2.36

**Table 8:** Mortality of Non-diapause Cephalcia chuxiongica Larvae Exposed to Different Concentrations of Phenylethyl Alcohol.

Treatment Time (h)	CK	0.05 mg/mL	0.5 mg/mL	5 mg/mL
	Mean ± SD			
12	0.00 ± 0.00	0.00 ± 0.00	3.33 ± 0.00	6.67 ± 1.67
24	0.00 ± 0.00	0.00 ± 0.00	3.33 ± 0.00	18.33 ± 8.82
36	0.00 ± 0.00	0.00 ± 0.00	3.33 ± 4.71	23.33 ± 8.82
48	0.00 ± 0.00	0.00 ± 0.00	6.67 ± 0.00	30.00 ± 5.77
60	5.00 ± 2.89	0.00 ± 0.00	6.67 ± 0.00	35.00 ± 5.00
72	5.00 ± 2.89	0.00 ± 0.00	20.00 ± 0.01	46.67 ± 4.41
84	5.00 ± 2.89	1.67 ± 1.67	21.67 ± 1.67	51.67 ± 6.67
96	5.00 ± 2.89	3.33 ± 1.67	28.33 ± 1.67	55.00 ± 7.64
108	8.33 ± 6.01	6.67 ± 1.67	31.67 ± 1.67	61.67 ± 6.01
120	10.00 ± 2.89	6.67 ± 1.67	31.67 ± 1.67	65.00 ± 5.77
132	10.00 ± 2.89	8.33 ± 3.33	35.00 ± 3.35	66.67 ± 4.41
144	10.00 ± 2.89	13.33 ± 1.67	55.00 ± 1.67	68.33 ± 6.01
156	10.00 ± 2.89	13.33 ± 1.67	60.00 ± 1.67	68.33 ± 6.01
168	20.00 ± 4.08	13.33 ± 1.67	68.33 ± 1.679	68.33 ± 6.01
180	20.00 ± 4.08	20.00 ± 4.08	76.67 ± 4.08	76.67 ± 17.00

**Table 9:** Mortality of Non-diapause Cephalcia chuxiongica Larvae Exposed to Different Concentrations of Benzeneacetic Acid.

Treatment Time (h)	CK	0.05 mg/mL	0.5 mg/mL	5 mg/mL
	Mean ± SD			
12	0.00 ± 0.00	0.00 ± 0.00	0.00 ± 0.00	20.00 ± 2.89
24	0.00 ± 0.00	0.00 ± 0.00	1.67 ± 2.36	23.33 ± 1.67
36	0.00 ± 0.00	0.00 ± 0.00	5.00 ± 4.08	23.33 ± 1.67
48	0.00 ± 0.00	0.00 ± 0.00	8.33 ± 4.71	25.00 ± 0.00
60	5.00 ± 2.89	3.33 ± 1.67	11.67 ± 2.36	56.67 ± 1.67
72	5.00 ± 2.89	3.33 ± 1.67	16.67 ± 2.36	61.67 ± 6.24
84	5.00 ± 2.89	5.00 ± 0.00	20.00 ± 4.08	100.00 ± 0.00
96	5.00 ± 2.89	5.00 ± 0.00	21.67 ± 4.71	100.00 ± 0.00
108	8.33 ± 6.01	6.67 ± 1.67	25.00 ± 4.08	100.00 ± 0.00
120	10.00 ± 2.89	8.33 ± 1.67	31.67 ± 4.71	100.00 ± 0.00
132	10.00 ± 2.89	10.00 ± 2.89	38.33 ± 6.24	100.00 ± 0.00
144	10.00 ± 2.89	10.00 ± 2.89	43.33 ± 6.24	100.00 ± 0.00
156	10.00 ± 2.89	10.00 ± 2.89	48.33 ± 4.08	100.00 ± 0.00
168	20.00 ± 4.08	13.33 ± 10.93	61.67 ± 6.24	100.00 ± 0.00
180	20.00 ± 4.08	13.33 ± 10.93	70.00 ± 4.08	100.00 ± 0.00

**Table 10:** Mortality of Non-diapause Cephalcia chuxiongica Larvae Exposed to Different Concentrations of Hydrocinnamic Acid.

Treatment Time (h)	CK	0.05 mg/mL	0.5 mg/mL	5 mg/mL
	Mean ± SD			
12	0.00 ± 0.00	0.00 ± 0.00	0.00 ± 0.00	6.67 ± 1.67
24	0.00 ± 0.00	0.00 ± 0.00	1.67 ± 2.36	18.33 ± 8.82
36	0.00 ± 0.00	0.00 ± 0.00	1.67 ± 2.36	23.33 ± 8.82
48	0.00 ± 0.00	0.00 ± 0.00	3.33 ± 2.36	30.00 ± 5.77
60	5.00 ± 2.89	0.00 ± 0.00	5.00 ± 4.08	35.00 ± 5.00
72	5.00 ± 2.89	0.00 ± 0.00	23.33 ± 5.00	46.67 ± 4.41
84	5.00 ± 2.89	1.67 ± 1.67	31.67 ± 6.01	51.67 ± 6.67
96	5.00 ± 2.89	3.33 ± 1.67	36.67 ± 6.67	55.00 ± 7.64
108	8.33 ± 6.01	5.00 ± 0.00	41.67 ± 4.41	58.33 ± 10.14
120	10.00 ± 2.89	6.67 ± 1.67	65.00 ± 8.82	61.67 ± 10.93
132	10.00 ± 2.89	6.67 ± 1.67	70.00 ± 8.82	63.33 ± 9.28
144	10.00 ± 2.89	8.33 ± 3.33	71.67 ± 7.64	65.00 ± 7.64
156	10.00 ± 2.89	8.33 ± 3.33	75.00 ± 8.82	66.67 ± 4.41
168	20.00 ± 4.08	8.33 ± 3.33	76.67 ± 6.01	68.33 ± 6.01
180	20.00 ± 4.08	10.00 ± 2.89	80.00 ± 6.67	68.33 ± 6.01

## Data Availability

Data from this trial can be found in the document and additional information package. Reagents, larvae and microbial materials, as well as data sets used, created, and analysed during this work, are available from the reporting author upon request.

## Supplementary Materials

The following supporting information can be downloaded at https://www.mdpi.com/article/10.3390/microorganisms13102289/s1, Table S1: Mortality of *Cephalcia chuxiongica* Larvae Exposed to Different Concentrations of Oleic Acid; Table S2: Mortality of *Cephalcia chuxiongica* Larvae Exposed to Different Concentrations of Tryptophol; Table S3: Mortality of *Cephalcia chuxiongica* Larvae Exposed to Different Concentrations of Stearic Acid; Table S4: Mortality of *Cephalcia chuxiongica* Larvae Exposed to Different Concentrations of Myristic Acid; Table S5: Mortality of *Cephalcia chuxiongica* Larvae Exposed to Different Concentrations of Dodecanoic Acid; Table S6: Mortality of *Cephalcia chuxiongica* Larvae Exposed to Different Concentrations of 2-Piperidinone; Table S7: Mortality of *Cephalcia chuxiongica* Larvae Exposed to Different Concentrations of Phenylethyl Alcohol; Table S8: Mortality of *Cephalcia chuxiongica* Larvae Exposed to Different Concentrations of Benzeneacetic Acid; Table S9: Mortality of *Cephalcia chuxiongica* Larvae Exposed to Different Concentrations of Hydrocinnamic Acid; Table S10: Mortality of *Cephalcia chuxiongica* Larvae Exposed to Different Concentrations of Conidial Suspension of Clonostachys rosea.
