# Supplementary material for: Analysis of Toxic Components in Secondary Metabolites of Entomopathogenic Fungi Clonostachys rosea (Hipocreales: Bionectriaceae) from Cephalcia chuxiongica (Hymenoptera: Pamphiliidae)
Source: Microorganisms. 2025 Oct 1;13(10):2289. doi: 10.3390/microorganisms13102289 (PMC12565781; doi:10.3390/microorganisms13102289)
Supplement: Supplementary file 1 [file microorganisms-13-02289-s001.zip › microorganisms-3799448-supplementary.pdf]

Table S1. Mortality of *Cephalcia chuxiongica* Larvae Exposed to Different Concentrations of Oleic Acid

Treatment time (h)	CK	0.05 mg/mL	0.5 mg/mL	5 mg/mL
	Mean ± SD			
12	0.00±0.00	0.00±0.00	5.00±4.08	43.33±15.9
24	0.00±0.00	0.00±0.00	5.00±4.08	65.00±5.77
36	0.00±0.00	0.00±0.00	15.00±10.8	65.00±5.77
48	0.00±0.00	0.00±0.00	26.67±6.24	78.33±1.67
60	5.00±2.89	0.00±0.00	30.00±7.07	90.0±2.89
72	5.00±2.89	1.67±1.67	31.67±8.5	95.00±2.89
84	5.00±2.89	3.33±1.67	41.67±4.71	98.33±1.67
96	5.00±2.89	3.33±1.67	41.67±4.71	100.00±0.00
108	8.33±6.01	6.67±1.67	51.67±4.71	100.00±0.00
120	10.00±2.89	6.67±1.67	65.00±4.08	100.00±0.00
132	10.00±2.89	10.00±2.89	71.67±4.71	100.00±0.00
144	10.00±2.89	10.00±2.89	76.67±7.07	100.00±0.00
156	10.00±2.89	10.00±2.89	78.33±8.5	100.00±0.00
168	20.00±4.08	10.00±2.89	78.33±8.5	100.00±0.00
180	20.00±4.08	20.00±4.08	81.67±9.43	100.00±0.00

Table S2. Mortality of *Cephalcia chuxiongica* Larvae Exposed to Different Concentrations of Tryptophol

Treatment time (h)	CK	0.05 mg/mL	0.5 mg/mL	5 mg/mL
	Mean ± SD			
12	0.00±0.00	0.00±0.00	11.67±6.24	50.00±5.00
24	0.00±0.00	0.00±0.00	11.67±6.24	53.33±4.41
36	0.00±0.00	0.00±0.00	13.33±8.50	53.33±4.41
48	0.00±0.00	1.67±1.67	21.67±2.36	60.00±0.00
60	5.00±2.89	3.33±1.67	25.00±4.08	63.33±1.67
72	5.00±2.89	5.00±0.00	36.67±10.27	68.33±4.41
84	5.00±2.89	5.00±2.89	41.67±9.43	68.33±4.41
96	5.00±2.89	6.67±1.67	46.67±9.28	85.00±2.89
108	8.33±6.01	10.00±2.89	51.67±4.71	86.67±1.67
120	10.00±2.89	13.33±4.41	56.67±4.71	86.67±1.67
132	10.00±2.89	15.00±2.89	66.67±7.64	88.33±1.67
144	10.00±2.89	15.00±2.89	68.33±5.77	88.33±1.67
156	10.00±2.89	15.00±2.89	65.00±5.77	90.00±2.89
168	20.00±4.08	15.00±2.89	73.33±2.89	95.00±2.89
180	20.00±4.08	15.00±2.89	75.00±2.89	98.33±1.67

Table S3. Mortality of *Cephalcia chuxiongica* Larvae Exposed to Different Concentrations of Stearic Acid

Treatment time (h)	CK	0.05 mg/mL	0.5 mg/mL	5 mg/mL
	Mean ± SD			
12	0.00±0.00	0.00±0.00	0.00±0.00	10.00±5.77
24	0.00±0.00	0.00±0.00	0.00±0.00	40.00±8.66
36	0.00±0.00	0.00±0.00	5.00±4.08	51.67±8.33
48	0.00±0.00	0.00±0.00	11.67±2.36	53.33±9.28
60	5.00±2.89	0.00±0.00	11.67±2.36	58.33±9.28

72	5.00±2.89	0.00±0.00	13.33±4.71	65.00±5.77
84	5.00±2.89	3.33±1.67	16.67±4.71	73.33±1.67
96	5.00±2.89	5.00±0.00	21.67±4.71	80.00±5.77
108	8.33±6.01	6.67±1.67	25.00±7.07	80.00±5.77
120	10.00±2.89	6.67±1.67	25.00±7.07	86.67±1.67
132	10.00±2.89	8.33±3.33	35.00±7.07	88.33±1.67
144	10.00±2.89	10.00±2.89	55.00±8.16	93.33±4.41
156	10.00±2.89	10.00±2.89	70.00±7.07	95.00±2.89
168	20.00±4.08	13.33±4.41	71.67±6.24	98.33±1.67
180	20.00±4.08	15.00±2.89	75.00±4.08	98.33±1.67

Table S4. Mortality of *Cephalcia chuxiongica* Larvae Exposed to Different Concentrations of Myristic Acid

Treatment time (h)	CK	0.05 mg/mL	0.5 mg/mL	5 mg/mL
	Mean ± SD			
12	0.00±0.00	0.00±0.00	5.00±0.00	10.00±2.89
24	0.00±0.00	0.00±0.00	5.00±0.00	25.00±5.00
36	0.00±0.00	0.00±0.00	10.00±4.08	35.00±7.64
48	0.00±0.00	0.00±0.00	15.00±4.08	35.00±7.64
60	5.00±2.89	0.00±0.00	23.33±4.71	53.33±1.67
72	5.00±2.89	0.00±0.00	25.00±4.08	60.00±2.89
84	5.00±2.89	1.67±1.67	35.00±4.08	71.67±9.28
96	5.00±2.89	3.33±1.67	45.00±4.08	86.67±3.33
108	8.33±6.01	6.67±1.67	48.33±2.36	90.00±5.00
120	10.00±2.89	8.33±3.33	51.67±2.36	95.00±2.89
132	10.00±2.89	10.00±5.00	56.67±4.71	96.67±3.33
144	10.00±2.89	11.67±4.41	66.67±7.64	100.00±0.00
156	10.00±2.89	11.67±4.41	68.33±1.67	100.00±0.00
168	20.00±4.08	13.33±10.93	73.33±6.24	100.00±0.00
180	20.00±4.08	13.33±10.93	73.33±6.24	100.00±0.00

Table S5. Mortality of *Cephalcia chuxiongica* Larvae Exposed to Different Concentrations of Dodecanoic Acid

Treatment time (h)	CK	0.05 mg/mL	0.5 mg/mL	5 mg/mL
	Mean ± SD			
12	0.00±0.00	0.00±0.00	1.67±2.36	25.00±8.66
24	0.00±0.00	0.00±0.00	1.67±2.36	33.33±4.41
36	0.00±0.00	0.00±0.00	3.33±2.36	31.67±8.82
48	0.00±0.00	0.00±0.00	8.33±8.50	53.33±18.78
60	5.00±2.89	0.00±0.00	18.33±2.36	60.00±16.07
72	5.00±2.89	0.00±0.00	18.33±2.36	61.67±17.40
84	5.00±2.89	1.67±1.67	20.00±4.08	68.33±11.67
96	5.00±2.89	3.33±1.67	31.67±1.67	81.67±6.01
108	8.33±6.01	3.33±1.67	31.67±1.67	91.67±1.67
120	10.00±2.89	5.00±2.89	35.00±3.35	96.67±1.67
132	10.00±2.89	10.00±2.89	38.33±13.12	96.67±1.67
144	10.00±2.89	10.00±2.89	43.33±6.24	96.67±1.67
156	10.00±2.89	11.67±2.36	48.33±4.08	100.00±0.00

168	20.00±4.08	11.67±2.36	55.00±1.67	100.00±0.00
180	20.00±4.08	20.00±4.08	60.00±1.67	100.00±0.00

Table S6. Mortality of *Cephalcia chuxiongica* Larvae Exposed to Different Concentrations of 2-Piperidinone

Treatment time (h)	CK	0.05 mg/mL	0.5 mg/mL	5 mg/mL
	Mean ± SD			
12	0.00±0.00	0.00±0.00	0.00±0.00	0.00±0.00
24	0.00±0.00	0.00±0.00	1.67±2.36	0.00±0.00
36	0.00±0.00	0.00±0.00	5.00±0.00	0.00±0.00
48	0.00±0.00	0.00±0.00	5.00±0.00	0.00±0.00
60	5.00±2.89	0.00±0.00	6.67±2.36	1.67±1.67
72	5.00±2.89	0.00±0.00	8.33±2.36	1.67±1.67
84	5.00±2.89	1.67±1.67	11.67±2.36	3.33±1.67
96	5.00±2.89	3.33±1.67	11.67±2.36	10.00±2.89
108	8.33±6.01	3.33±1.67	16.67±2.36	10.00±2.89
120	10.00±2.89	3.33±1.67	16.67±4.71	10.00±2.89
132	10.00±2.89	3.33±1.67	16.67±4.71	10.00±2.89
144	10.00±2.89	3.33±1.67	20.00±0.01	10.00±2.89
156	10.00±2.89	5.00±2.89	20.00±0.01	11.67±2.36
168	20.00±4.08	5.00±2.89	21.67±1.67	11.67±2.36
180	20.00±4.08	8.33±6.01	21.67±1.67	16.67±2.36

Table S7. Mortality of *Cephalcia chuxiongica* Larvae Exposed to Different Concentrations of Phenylethyl Alcohol

Treatment time (h)	CK	0.05 mg/mL	0.5 mg/mL	5 mg/mL
	Mean ± SD			
12	0.00±0.00	0.00±0.00	3.33±0.00	6.67±1.67
24	0.00±0.00	0.00±0.00	3.33±0.00	18.33±8.82
36	0.00±0.00	0.00±0.00	3.33±4.71	23.33±8.82
48	0.00±0.00	0.00±0.00	6.67±0.00	30.00±5.77
60	5.00±2.89	0.00±0.00	6.67±0.00	35.00±5.00
72	5.00±2.89	0.00±0.00	20.00±0.01	46.67±4.41
84	5.00±2.89	1.67±1.67	21.67±1.67	51.67±6.67
96	5.00±2.89	3.33±1.67	28.33±1.67	55.00±7.64
108	8.33±6.01	6.67±1.67	31.67±1.67	61.67±6.01
120	10.00±2.89	6.67±1.67	31.67±1.67	65.00±5.77
132	10.00±2.89	8.33±3.33	35.00±3.35	66.67±4.41
144	10.00±2.89	13.33±1.67	55.00±1.67	68.33±6.01
156	10.00±2.89	13.33±1.67	60.00±1.67	68.33±6.01
168	20.00±4.08	13.33±1.67	68.33±1.679	68.33±6.01
180	20.00±4.08	20.00±4.08	76.67±4.08	76.67±17.00

Table S8. Mortality of *Cephalcia chuxiongica* Larvae Exposed to Different Concentrations of Benzeneacetic Acid

Treatment time (h)	CK	0.05 mg/mL	0.5 mg/mL	5 mg/mL
	Mean ± SD			
12	0.00±0.00	0.00±0.00	0.00±0.00	20.00±2.89

24	0.00±0.00	0.00±0.00	1.67±2.36	23.33±1.67
36	0.00±0.00	0.00±0.00	5.00±4.08	23.33±1.67
48	0.00±0.00	0.00±0.00	8.33±4.71	25.00±0.00
60	5.00±2.89	3.33±1.67	11.67±2.36	56.67±1.67
72	5.00±2.89	3.33±1.67	16.67±2.36	61.67±6.24
84	5.00±2.89	5.00±0.00	20.00±4.08	100.00±0.00
96	5.00±2.89	5.00±0.00	21.67±4.71	100.00±0.00
108	8.33±6.01	6.67±1.67	25.00±4.08	100.00±0.00
120	10.00±2.89	8.33±1.67	31.67±4.71	100.00±0.00
132	10.00±2.89	10.00±2.89	38.33±6.24	100.00±0.00
144	10.00±2.89	10.00±2.89	43.33±6.24	100.00±0.00
156	10.00±2.89	10.00±2.89	48.33±4.08	100.00±0.00
168	20.00±4.08	13.33±10.93	61.67±6.24	100.00±0.00
180	20.00±4.08	13.33±10.93	70.00±4.08	100.00±0.00

Table S9. Mortality of *Cephalcia chuxiongica* Larvae Exposed to Different Concentrations of Hydrocinnamic Acid

**Treatment time (h)**	**CK**	**0.05 mg/mL**	**0.5 mg/mL**	**5 mg/mL**
	**Mean ± SD**			
12	0.00±0.00	0.00±0.00	0.00±0.00	6.67±1.67
24	0.00±0.00	0.00±0.00	1.67±2.36	18.33±8.82
36	0.00±0.00	0.00±0.00	1.67±2.36	23.33±8.82
48	0.00±0.00	0.00±0.00	3.33±2.36	30.00±5.77
60	5.00±2.89	0.00±0.00	5.00±4.08	35.00±5.00
72	5.00±2.89	0.00±0.00	23.33±5.00	46.67±4.41
84	5.00±2.89	1.67±1.67	31.67±6.01	51.67±6.67
96	5.00±2.89	3.33±1.67	36.67±6.67	55.00±7.64
108	8.33±6.01	5.00±0.00	41.67±4.41	58.33±10.14
120	10.00±2.89	6.67±1.67	65.00±8.82	61.67±10.93
132	10.00±2.89	6.67±1.67	70.00±8.82	63.33±9.28
144	10.00±2.89	8.33±3.33	71.67±7.64	65.00±7.64
156	10.00±2.89	8.33±3.33	75.00±8.82	66.67±4.41
168	20.00±4.08	8.33±3.33	76.67±6.01	68.33±6.01
180	20.00±4.08	10.00±2.89	80.00±6.67	68.33±6.01

Table S10. Mortality of *Cephalcia chuxiongica* Larvae Exposed to Different Concentrations of Conidial Suspension of *Clonostachys rosea*

**Treatment time (h)**	**CK**	Conidial suspension of *Clonostachys rosea*
	**Mean ± SD**	
12	0.00±0.00	0.00±0.00
24	0.00±0.00	15.00±0.00
36	0.00±0.00	15.00±0.00
48	0.00±0.00	20.00±2.89
60	5.00±2.89	30.00±2.89
72	5.00±2.89	46.67±7.26
84	5.00±2.89	63.33±9.28
96	5.00±2.89	83.33±1.67
108	8.33±6.01	88.33±1.67

120	10.00±2.89	91.67±1.67
132	10.00±2.89	96.67±1.67
144	10.00±2.89	98.33±1.67
156	10.00±2.89	100.00±0.00
168	20.00±4.08	100.00±0.00
180	20.00±4.08	100.00±0.00
